# Shear Instability and Localization in High-Speed Cold Spray Processes: Impact on Particle Fragmentation and Bonding Mechanisms

**DOI:** 10.3390/ma18030490

**Published:** 2025-01-22

**Authors:** Sabeur Msolli

**Affiliations:** ICB UMR 6303 CNRS, Université Marie et Louis Pasteur, UTBM, 90010 Belfort Cedex, France; sabeur.msolli@utbm.fr

**Keywords:** cold spray, dislocation density, adiabatic shear instability, adiabatic shear localization, particle fragmentation, bonding

## Abstract

This study investigates the deformation behavior and interfacial phenomena occurring during the high-velocity impact of a copper particle into a copper substrate under various conditions using FEM. It also offers an enhanced physics-based model based on discrete dislocation dynamics simulations to depict newly observed features such as interfacial instabilities and shear localization leading to bonding and particle fragmentation. To investigate bonding mechanisms at the particle–substrate interface, additional simulations using a one-element-thickness model are conducted. These simulations focus on the deformation behavior at the interface, revealing wavy shape formation in the substrate due to disparities in strain-rate levels. Material instabilities, localized at the intersection of plane and release waves, progress hand-in-hand during the early stages of impact, suggesting shear behavior as a precursor to instabilities. The effect of shear viscosity on particle deformation and interfacial behavior is also examined, showing that increased viscosity leads to thermal material softening and enhanced deformation. Material jetting and interfacial instability are observed, particularly at higher viscosity thresholds. Additionally, the impact of drag coefficient variations on particle deformation is explored, indicating a critical role in interfacial stability and particle flattening. Finally, the occurrence of adiabatic shear instability and localization is investigated, revealing shear localization regions at the particle–substrate interface and within the particle itself responsible for particle fragmentation. To this aim, damage initiation and evolution laws are applied to identify regions of shear localization, crucial for particle–substrate bonding and mechanical interlocking. The impact velocity is shown to influence shear localization, with higher velocities resulting in increased deformation and larger localization regions.

## 1. Introduction

The aerospace and aeronautics industries are placing considerable emphasis on the bonding strength of cold-sprayed metallic coatings onto metallic substrates. With a growing demand for lightweight structures, the cold-spray process has emerged as an exceptional solution for crafting coatings of varying thicknesses from a wide range of material combinations. Additionally, it proves invaluable for the intricate task of repairing geometrically complex damaged parts. The coatings achieved through cold spray are not only expected to exhibit outstanding mechanical properties and a minimal density of defects but also to establish a robust bond with the substrate. This bond strength and the mechanical attributes of the coating are intricately linked to several process parameters, including particle diameter, substrate surface roughness, deposition pressure, material combination, feeding rate, gas properties (nature and temperature), as well as the size distribution of the powder, etc [1,2,3,4,5]. These factors collectively contribute to generating specific impact conditions. The impact of the powder onto the target substrate is primarily determined by the impact velocity and angle, as well as the powder temperature. These parameters have a direct impact on the final mechanical properties of the produced coating [3,4,6,7,8,9]. Among the critical factors for assessing coating quality are the strength properties, defect density, and bonding strength. The mechanical properties of the coating typically differ significantly from its original powder state [10,11]. This is because the powder undergoes high-velocity impact on the target substrate, leading to extreme plastic deformation, sometimes reaching up to 1000% in many deposition scenarios, with temperatures approaching, if not reaching, the melting point. As a result, profound microstructural changes occur, including phenomena like dislocation arrangement, networking, dynamic recrystallization, and subsequent grain refinement, which are quite common. Despite the excellent mechanical properties, defects like voids and cracks may be observed after the cold-spray process under specific deposition conditions, potentially compromising the integrity of the assemblies [6,10,12,13,14,15]. As these defects are undesirable in the final coating structure, there is a need to understand the relationship between process parameters and the occurrence of defects within the coating and at the coating–substrate interface. Several previous studies have been conducted to explore this relationship [13,16,17].

The high bonding strength of the coating is crucial for extending the lifespan of the cold-sprayed assembly. This forms the cornerstone of a comprehensive strategy to determine the bonding strength of cold-sprayed coatings, which is the primary motivation of this paper. The bonding strength is directly correlated with the impact properties [18,19]. For example, previous experimental and numerical findings have demonstrated that reducing the spray angle enhances the adhesion of the coating to the substrate, leading to the formation of dimple-like features at the particle–substrate interface [18]. It has also been shown that the quality of the surfaces involved during the impact significantly influences the bonding strength, and this bonding mechanism is governed by a solid-state process. Surface roughness and preparation prior to deposition have been identified as major factors in ensuring a strong interface bond [20,21]. According to numerous studies, attaining a critical velocity is crucial for achieving a robust bond between the substrate and the deposition material [22,23,24]. The elucidation of bonding mechanisms has been a longstanding endeavor, and various fundamental theories have emerged to explain how bonding occurs at the coating–substrate interface. A significant body of research has proposed adiabatic shear instability phenomena as the primary mechanism for bonding [19,25,26,27]. In situations of severe plastic deformation during impact at critical velocity, materials can undergo shear localization, resulting in material softening. This material softening at the coating–substrate interface promotes plastic flow instabilities. At this stage, the temperature is high, and the softened materials at the interface act as a fluid medium with varying dynamic viscosities. The dynamics of fluids with different properties in contact with each other can be explained by the Kelvin–Helmholtz Instability (KHI). This leads to the formation of irregular structures such as rollups and vortices, increasing the contact surface area and promoting mechanical interlocking. Additionally, the interfacial jet produced at the particle–substrate interface enhances surface cleaning and facilitates intimate contact between the particle and the substrate. In terms of quantification, the evaluation of the bonding strength of cold-sprayed coatings on substrates is typically conducted through standard experiments, supported by numerical simulations. Experiments primarily involve pull-off adhesion tests, such as ASTM standards C633–13 or D4541–02, among other variants. Many existing experimental setups include an intermediate glue layer to provide an estimate of the bond strength between the coating and the substrate. Recently, glue-free methods have emerged, reducing uncertainties associated with the use of adhesive materials [28]. Predicting the bonding strength of the coating numerically remains challenging. This is due to the significantly altered microstructure of the coating material before the adhesion test, the presence of randomly distributed voids and cracks within the coating, and the difficulty in predicting the coating–substrate interface numerically. Therefore, numerous publications have focused on understanding the bonding origin through the study of the particle–substrate interface at impact [18,21,22,26,29,30]. Many of these studies have addressed separate components of the problem without considering it holistically. Metallic materials not only exhibit different behavior at very high strain rates due to phonon drag, but also undergo drastic changes in microstructure, including dislocation density and grain size. Currently, there is no comprehensive numerical study correlating these microstructural characteristics with particle bonding. Many existing models incorporate only a few or even none of the relevant physics behind these microstructure evolutions [22,31,32,33]. Furthermore, while temperature is commonly considered a key indicator of particle bonding, it is believed that excessive hydrostatic pressure generated during impact also plays a crucial role in creating instabilities at the particle–substrate interface. Unfortunately, to the best of the authors’ knowledge, there is a lack of precise bonding strength data for a single particle on a substrate that could be used to identify the key bonding mechanisms and compare them with numerical simulations. In this paper, we do not present the entire methodology for estimating bonding strength. Instead, we aim to provide a comprehensive understanding of the particle–substrate bonding mechanism by considering the effects of very high strain rate, dislocation mechanics, and the extreme hydrostatic pressure at the particle impact. This study represents a preliminary step towards developing the right methodology for estimating the bonding strength of cold-sprayed coatings. In the limited available literature addressing additive manufacturing-related processes, material printing and behavior are typically modeled using specific tools.

## 2. Numerical Framework

### 2.1. Material Elasticity

In scenarios of shock, the strain rates experienced are exceptionally high. Consequently, two shock waves, one elastic and the other plastic, are generated and travel at different velocities. The plastic shock waves can be of such high intensity that they induce significant pressure, causing the material to approach or even exceed its yield strength under shock-loading conditions. In order to accurately model this hydrodynamic behavior of the material under extreme strain rates, we employed the equation of state (EOS), which offers a comprehensive account of how the elastic limit depends on the generated pressure. Addressing the abrupt changes in pressure, density, and internal energy that occur over an extremely short duration at the wavefront, various formulations have been proposed in the literature. For our study, we opted to utilize the Mie–Grüneisen equations of state [34], which establish a relationship between pressure and density as

(1) $$p=p_{H}\left(1-\frac{{\left(1-\frac{\rho_{0}}{\rho_{t}}\right)\Gamma }_{0}}{2}\right)+{\rho_{0}\Gamma }_{0}E_{m},$$

where $\Gamma_{0}$ is a material constant, $\rho_{t}$ is the current density, and $\rho_{0}$ is the reference density. $p_{H}$ and $E_{m}$ are the Hugoniot pressure and the increase in internal energy (per unit mass). $p_{H}$ is given by a fit of the Hugoniot data as

(2) $$p_{H}=\frac{\rho_{0}c_{0}^{2}\left(1-\frac{\rho_{0}}{\rho_{t}}\right)}{{\left(1-s_{c}\left(1-\frac{\rho_{0}}{\rho_{t}}\right)\right)}^{2}}$$

The linear Hugoniot form permits us to obtain $c_{0}$ and $s_{c}$ which define the linear relationship between the shock velocity $U_{s}$ and the particle velocity $U_{p}$

(3) $$U_{s}=c_{0}+s_{c}U_{p}$$

For austenitic steels, we adopted $c_{0}=4940$, $s_{c}=1.49$ and $\Gamma_{0}=1.93$. The increase in the internal energy $E_{m}$ is given by the following differential equation, which is solved iteratively by the FE solver at each iteration at each material point

(4) $$\rho_{t}\frac{dE_{m}}{dt}=\left(p-p_{bv}\right)\frac{1}{\rho_{t}}\frac{\partial \rho_{t}}{\partial t}+s:\dot{e}+\rho_{t}\dot{Q}$$

$p_{bv}$ is the pressure stress due to the bulk viscosity, s is the deviatoric stress tensor, $\dot{e}$ is the deviatoric part of strain rate, and $\dot{Q}$ is the heat rate per unit mass.

### 2.2. Kinetics of Plastic Deformation

When metallic particles impact metallic substrates, they induce microstructural alterations. The extreme plastic deformation leads to temperature-dependent processes such as dislocation multiplication and annihilation, dynamic recrystallization, and grain refinement. In cold-spray applications, it is common to observe the presence of ultra-fine grains (UFG) measuring significantly less than 1 μm at the interface between the coating and substrate, as well as in its immediate vicinity [35,36,37]. An increase in the misorientation angle is noted due to the accumulation of dislocations along cell walls and grain boundaries [38,39,40,41]. Cell substructures have also been identified using Transmission Electron Microscopy (TEM). Given these experimental observations, it is logical to employ a physics-based plasticity model to accurately depict the evolution of these inherent properties. A widely used physics-based plasticity model for processes involving extreme plastic deformation, including impact scenarios [6,40,42,43,44,45], is the dislocation density-based model developed by Estrin et al., [46,47,48,49]. This model comprises a set of equations that describe the relationship between material deformation and the density of dislocations. It takes into account the reduction in cell size as the dislocation density increases [50]

(5) $$d=\frac{K}{\sqrt{\rho }}$$

where *K* is a strain-dependent parameter, which evolves with the resolved shear strain as the following

(6) $$K=K_{\infty }+\left(K_{0}-K_{\infty }\right)exp\left(-\beta \gamma_{r}\right)$$

$K_{0}$ is the initial value of the parameter *K*. $K_{\infty }$ is the saturated value reached with the accumulation of the dislocation density, $\gamma^{r}$ is the resolved shear strain, and β is a parameter. Holt’s Equation (5) should be considered with caution as it is applied to low-to-moderate strain-rate scenarios. The primary results in [51] showed trends of numerically predicted grain size with an overall good agreement with those experimentally measured.

The total dislocation density ρ is the weighted sum of dislocations in the cell interior $\rho_{I}$ and piled-up dislocations at the cell wall $\rho_{w}$, corrected with the density of dislocations $\rho_{CID}$ accumulated in the grain boundary generated from the lattice curvature due to the constraining of the neighbor grains [52,53]. Whereas cell interior dislocations correspond to dislocations embedded in the cell and are susceptible to motion until reaching obstacles or defects, cell-wall dislocations are those that pile up forming barriers or tangles delimiting the dislocation cell size. The total dislocation density is expressed as

(7) $$\rho =f\rho_{w}+\left(1-f\right)\rho_{I}+\rho_{CID}$$

As for *K*, the volume fraction of the dislocation walls *f* obeys an equivalent form where a saturated value is reached for high plastic strain. That means

(8) $$f=f_{\infty }+\left(f_{0}-f_{\infty }\right)exp\left(-\gamma_{r}/\hat{\gamma }\right)$$

$f_{0}$ and $f_{\infty }$ are the initial and saturated volume fraction of the dislocation walls, respectively. The $\hat{\gamma }$ is a material constant that controls the rate of reduction in f. The density of the two types of dislocation moving in the cell wall and cell interior is calculated with the following partial differential equations

(9) $${\dot{\rho }}_{I}=\alpha^{*}\frac{1}{\sqrt{3}}\frac{\sqrt{\rho_{w}}}{b}{\dot{\gamma }}_{w}-\beta^{*}\frac{6{\dot{\gamma }}_{I}}{bd{\left(1-f\right)}^{\frac{1}{3}}}-{\dot{\gamma }}_{I}k_{I}{\left(\frac{{\dot{\gamma }}_{I}}{{\dot{\gamma }}_{0}}\right)}^{-T/A}\rho_{I}$$

(10) $${\dot{\rho }}_{w}={\dot{\rho }}_{ws}+{\dot{\rho }}_{wg}=\beta^{*}\frac{6{\dot{\gamma }}_{I}{\left(1-f\right)}^{\frac{2}{3}}}{bdf}{+\beta }^{*}\frac{\sqrt{3}{\dot{\gamma }}_{w}\left(1-f\right)\sqrt{\rho_{w}}}{fb}-{\dot{\gamma }}_{w}k_{w}{\left(\frac{{\dot{\gamma }}_{w}}{{\dot{\gamma }}_{0}}\right)}^{-T/A}\rho_{w}$$

where *A* is a constant, *T* is the temperature in a unit (K), and $k_{I}$ and $k_{w}$ are temperature-dependent functions driving the dynamic recovery [43]. They can be expressed as polynomials in the following form, accounting for the material parameters $k_{i1}$, $k_{i2}$, and $k_{i3}$:

(11) $$k_{i}=k_{i1}T^{2}+k_{i2}T+k_{i3}\;\;\;\;\;\;(i=i,w)$$

where n is a strain-rate sensitivity parameter, ${\dot{\gamma }}_{0}$ is a reference shear rate, and b is the magnitude of Burgers vector. The shear strain rates for cell interiors ${\dot{\gamma }}_{I}$, and cell walls ${\dot{\gamma }}_{w}$ are assumed to be equal to the resolved shear strain rate ${\dot{\gamma }}_{r}$. The resolved plastic shear strain $\gamma_{r}$ is related to the macroscopic strain *ε* through Taylor parameter *M*, which is assumed constant in the present study and equal to 3.06. $\alpha^{*}$ is the fraction of Frank–Read sources of the interface operated by the wall dislocations. $\beta^{*}$ is the fraction of dislocations that migrate from the cell interior to the cell wall. Note that the initial values of $\rho_{I}$ and $\rho_{w}$ are $\rho_{I0}$ and $\rho_{w0}$, respectively. As can be seen from Equations (9) and (10), the evolution of the dislocation densities are monitored by a competition between the generation of dislocations due to the activation of Frank–Read sources, the transfer of cell interior dislocations to cell walls, and the annihilation of dislocations due to dynamic recovery. The cell-wall dislocation density is considered as the sum of a statistical dislocation density $\rho_{ws}$ and a geometrically necessary dislocation density $\rho_{wg}$, which are due to cell misorientations where

(12) $${\dot{\rho }}_{ws}=\left(1-\omega_{1}\right)\beta^{*}\frac{6{\dot{\gamma }}_{I}{\left(1-f\right)}^{\frac{2}{3}}}{bdf}{+\left(1-\omega_{2}\right)\beta }^{*}\frac{\sqrt{3}{\dot{\gamma }}_{I}\left(1-f\right)\sqrt{\rho_{w}}}{fb}\;-k_{0}{\dot{\gamma }}_{I}{\left(\frac{{\dot{\gamma }}_{w}}{{\dot{\gamma }}_{0}}\right)}^{-T/A}\rho_{ws}$$

and

(13) $${\dot{\rho }}_{wg}=\omega \beta^{*}\frac{6{\dot{\gamma }}_{I}{\left(1-f\right)}^{\frac{2}{3}}}{bdf}+{+\omega_{2}\beta }^{*}\frac{\sqrt{3}{\dot{\gamma }}_{I}\left(1-f\right)\sqrt{\rho_{w}}}{fb}$$

where $\omega_{1}$ and $\omega_{2}$ are the fractions of the contribution of the geometrically necessary dislocation and the statistical dislocation on the dislocation wall density, respectively. The geometrically necessary dislocation density measured using the EBSD technique is not restricted to cell misorientation contribution only, including curvature-induced dislocation density as well. The difference is that the first is difficult to establish because the cell misorientation is relatively small. Nevertheless, they are accounted for in the measurement of the GNDs using EBSD through the calculation of the dislocation density tensor [54]. The contribution of the lattice curvature into the dislocation density depends on the number k of the neighboring grains in contact with the deformed grain, and the angle Ω formed by the lattice curvature due to the constrained contact with these grains [52]. It was previously shown that the particles of the cold-spray metal powder are likely to present as single monocrystals or single grains with small satellite grains [38]. In this case, we choose a parameter *k* equal to 1, where *k* is introduced to obtain the total density of CIDs when curvature is applied in three different directions according to the number of grain boundaries (GB) adjacent locally to the GB zone (one, two, or three neighboring grains) [52]:

(14) $$\rho_{CID}=k\frac{12sin\left(\mu \Omega \right)}{bD_{p}\left(-cos\left(\mu \Omega \right)+\sqrt{{cos}^{2}\left(\mu \Omega \right)+8}\right)}$$

μ is a coefficient equal to 0.1. It is then understood that the geometrically necessary dislocation density obtained by EBSD is nothing but $\rho_{GND}=\rho_{CID}+\rho_{wg}$. Now, the deformation at a high strain rate of materials crosses the dislocation drag-dominated regime. Through recent discrete dislocation dynamic simulations, it was shown that at a wide regime of high strain rates, the material yield stress can vary significantly, not only with the strain rate but also with the initial dislocation density [55]. Then, yield stress increase is a contribution of both strain-rate hardening and forest dislocation hardening [56]. Inspired by these modifications, we brought these considerable changes to the expression of the resolved shear stress. In addition, we introduced the linear strain-rate sensitivity *s* relevant for the plastic regime to express the flow stress $\sigma_{y}$ [55]:

(15) $$\sigma_{y}=s\left(T,\rho \right)\dot{\varepsilon }+M\alpha G\left(T\right)b\sqrt{\rho }$$

where α is a constant, and G is the temperature-dependent shear modulus. In the present context of exasperated deformation, G obeys the MTS (Mechanical Threshold Stress) shear modulus form as written in [57,58]

(16) $$G\left(T\right)=G_{0}-\frac{D}{\exp\left(\frac{T_{0}}{T}\right)-1}$$

where $G_{0}$ is the shear modulus at 0 K, which is equal to 58.1 GPa for copper, and D and $T_{0}$ are material constants equal to 3 GPa and 165 K, respectively. An expression of the strain-rate sensitivity s is proposed in [55,59,60], and can be written as:

(17) $$s=\frac{M^{2}B}{\rho b^{2}}$$

Equation (15) is involved in both yielding and plasticity regimes. At yield, it degenerates to the expression obtained from DDD (Discrete Dislocation Dynamics) simulations. Therefore, the results of the dependency of the yield stress to strain rate and dislocation density can be obtained ($\rho =\rho_{0}$). When the material engages on the plastic regime, then the expression of the resolved shear stress evolves according to the evolving plastic deformation and hence to the updated dislocation density. The strain-rate sensitivity increases with temperature and this is captured through the temperature-dependent drag coefficient B. Unfortunately, the drag coefficient for copper is poorly reported and the few experimental and molecular dynamics studies on this coefficient were performed at low temperatures up to 400 K [61,62]. As such, the use of Leibfried’s estimate is of great help in this present case study to obtain an estimate at extreme temperature up to melting temperature [63,64]. We adopted the following linear relationship according to [65]:

(18) $$B(Pa.s)=B_{0}T-B_{1}$$

The parameters $B_{0}$ and $B_{1}$ are $1.09\times 10^{-7}Pa.s.T^{-1}$ and ${1.2\times 10}^{-5}Pa.s$, respectively. The drag is a very critical in deciding the influence of the high speed impact and temperature on the particle deformation. To show the corresponding effect, many case studies are presented in the following sections through varying B by a factor of 10, 100 and 1000.

A misorientation angle is computed based on the fact that the GNDs mostly contribute to the misorientation across the cell wall. Accordingly, the misorientation angle is calculated as follows

(19) $$\theta_{mis}=Arctan\left(b\sqrt{\rho_{wg}}\right)$$

As the increase in the misorientation angle enhances the movement of the produced dislocations from the cell interior to the cell wall, the parameter $\beta^{*}$ monitoring the evolution rate of the wall dislocation density increases. This relationship turns into

(20) $$\;\;\beta^{*}=\beta_{\infty }+\left(\beta_{0}-\beta_{\infty }\right)exp\left(-\frac{\theta_{mis}}{\pi /12}\right)$$

Here, $\beta_{0},\beta_{\infty }$ are the initial and saturated values of $\beta^{*}$, respectively.

At a regime of a high temperature approaching melting, the material behaves more as a fluid-like media. To account for this in the presented model, we preserved the assumption of a Newtonian material with a shear viscosity $\mu_{v}$ at a temperature higher than a certain threshold. Accordingly, the flow stress becomes purely linearly dependent on the strain rate, as follows:

(21) $$\sigma =\mu_{v}\dot{\varepsilon }$$

The shear viscosity of copper at a temperature near melting temperature (≈5T/6), beyond (1600 K), and in the relevant range of the strain rate proper to the present application (1/s to 1012/s) is determined from molecular dynamics using a many-body embedded-atoms model potential and reported in [66]. A resultant relationship is obtained below:

(22) $$\mu_{v}(Pa.s)=\mu_{1}T^{2}-\mu_{2}T+\mu_{3}$$

The parameters $\mu_{1}$, $\mu_{2}$ and $\mu_{3}$ are $3.79\times 10^{-9}Pa.s.T^{-2}$, $1.38\times 10^{-5}Pa.s.T^{-1}$, and $1.53\times 10^{-2}Pa.s$, respectively. The thermal properties of copper are given in Table 1.

### 2.3. Adiabatic Shear Instability and Shear Localization

Adiabatic shear instability occurs when material softening exceeds strain hardening, leading to localized shear deformation. This phenomenon is influenced by factors such as strain rate and temperature rise. While stress–strain curves may exhibit sudden drops under adiabatic conditions, adiabatic shear instability involves rapid shear band formation due to localized heating and deformation. In an ideal case, this material softening occurs homogeneously inside the material. However, in reality, there are fluctuations of the state variables and microstructural properties, and geometrical imperfections leading to shear localization. In processes like cold spray, where particle impacts are brief, adiabatic shear instability may not necessarily occur within the impact duration alone. Its onset is influenced by various factors beyond impact duration, including material properties and process parameters [25]. Typical examples include but are not limited to necking, bulging, and at a smaller scale, the formation of shear bands, vortices, and rollups. There is also a significant strain and thermal gradient between the region of the shear localization and the surrounding media, causing a quick drop in the flow stress [67]. Understanding the onset of adiabatic shear instability is crucial for predicting shear localization and assessing particle–substrate bonding mechanisms. Various theories have been developed to understand this phenomenon, though it is not strictly a post-stage or bifurcation phenomenon [68,69,70,71,72,73,74,75,76]. A comprehensive review of the shear instability works can be found in [67] for instance. In the following section, we derive a shear instability criterion tailored to our plasticity model to aid in determining the onset of localization. This derivation builds upon methodologies found in the relevant literature [77,78,79,80,81,82], among many others. The model consists of the following governing equations and initial conditions. With a simple departure equation assuming the explicit dependency of the flow stress to the strain, strain rate, and temperature, one can write:

(23) $$\sigma =f\left(\varepsilon ,\dot{\varepsilon },T\right)$$

As previously mentioned, a perturbation in strain, strain rate, or temperature will generate a fluctuation dσ that can be expressed as follows

(24) $$d\sigma =\frac{\partial f}{\partial \varepsilon }d\varepsilon +\frac{\partial f}{\partial \dot{\varepsilon }}d\dot{\varepsilon }+\frac{\partial f}{\partial T}dT$$

The deformation is homogeneous as long as dσ is positive. Shear instability occurs when dσ becomes negative, which is equivalent to a negative slope of the stress–strain curve. In adiabatic conditions, the formation of the shear bands becomes more prevalent at high strain rates because the thermal softening is significant with temperature increase. In these conditions, the plastic work can be converted to heat and the temperature increase reads

(25) $$dT=\frac{\xi \sigma d\varepsilon }{\varsigma C_{p}}$$

where ξ is the Taylor–Quinney factor, ς is the density, and $C_{p}$ is the specific heat of the material. Associating Equation (23) with (22) leads to

(26) $$d\sigma =\left(\frac{\partial \sigma }{\partial \varepsilon }+\frac{\partial \sigma }{\partial T}\frac{\xi \sigma }{\varsigma C_{p}}\right)d\varepsilon +\frac{\partial \sigma }{\partial \dot{\varepsilon }}d\dot{\varepsilon }$$

A detailed mathematical development has been presented for instance in [79] for the establishment of an instability criterion from Equation (23). In the present lines, we directly give the resultant criterion, which initially reads:

(27) $$V=-\frac{\frac{\partial \sigma }{\partial \varepsilon }+\frac{\partial \sigma }{\partial T}\frac{\xi \sigma }{\varsigma C_{p}}}{\frac{\partial \sigma }{\partial \dot{\varepsilon }}+\frac{\lambda }{C_{p}}}>0$$

where λ is the thermal conductivity. As the problems of particle impact on the substrate involve drastic microstructure changes in terms of dislocation density, one may expect the formation of shear instabilities to be tied to the dislocation density-based mechanism underlying the severe deformation along with the particle–substrate interface. Hence, the derivatives of Equation (25) can be obtained from Equation (14) and one obtains the following expression of the instability criterion:

(28) $$\;V=-\frac{\left(\frac{\alpha bG\sqrt{\rho }}{2}-\frac{MB\dot{\varepsilon }}{b^{2}\rho }\right)\frac{\left(\rho_{w}-\rho_{I}\right)\left(f_{\propto }-f\right)}{\hat{\gamma }\rho }+\left(\frac{B_{0}\dot{\varepsilon }}{b^{2}\rho }+\alpha bG_{0}\sqrt{\rho }\right)\frac{\xi \sigma }{\varsigma C_{p}}}{\frac{M^{2}B}{b^{2}\rho }+\frac{\lambda }{C_{p}}}>0$$

which we can readily apply to the prediction of the shear instability in the subsequent particle impact simulations. In an adiabatic shear localization regime, shear localization is likely to occur due to shear banding at the particle–substrate interface. For a particle that impinges the substrate at relatively low velocity, the amount of kinetic energy converted to plastic work may be enough to generate shear instability but insufficient to generate these shear bands. Contrarily, cracks crossing the particle or the substrate at the interface can be unavoidable. From a certain velocity, where the temperature at the interface is significant, the dynamic viscosity of the materials becomes high enough that shear banding can be associated with large deformation, resulting in the formation of specific features such as rollups. Shear localization has been a subject of interest for decades. The understanding of shear localization and the development of associated criteria for its onset was largely investigated. The onset of shear localization may be considered as the moment when the difference between the critical and maximal shear strain of the stress–strain curve becomes small enough, less than a threshold value, to trigger shear localization. The present work adopts this definition based on the works of Schoenfeld and Wright, who derived a shear localization factor [82] based on the following

(29) $$SL=-\frac{\frac{\partial^{2}\sigma }{\partial \varepsilon^{2}}}{\sigma \frac{\partial \sigma }{\partial \dot{\varepsilon }}}$$

For metals such as aluminum and copper, the shear localization factor has been found to take a value of 1.6 × 10^−4^ s/GPa ± 0.2 × 10^−4^ s/GPa based on numerous simulations [19,25,27].

### 2.4. Identification of Material Parameters

The material parameters which require calibration are principally those controlling the dislocation network changes. The calibration is based on experimental data of quasi-static and dynamic compression tests performed on copper samples at different temperatures and strain rates. Fortunately, the literature is rich in relevant databases of copper at the spectral of extreme loading conditions, which are valuable for the present framework. For instance, databases of copper can be found in [58,83,84,85,86] among many others. We underwent material calibration from stress and plastic strain curves. For the determination of the plastic deformation, J2-plasticity is used to calculate the Von Mises yield condition. The final outcome of the calibration is to find the minimum of an unconstrained residual function using the derivative-free method, i.e., the simplex search method of [87]. The residual function to be minimized is written as the following

(30) $$Residual=\frac{1}{N_{d}}\sum_{j=1}^{N_{d}}\frac{1}{N_{p}}\sqrt{\sum_{i=1}^{Np}{\left({\frac{\left({\sigma_{exp}}_{i}-{\sigma_{num}}_{i}\;\right)}{{\sigma_{exp}}_{i}^{2}}}^{2}\right)}_{j}}$$

**Figure 1:**
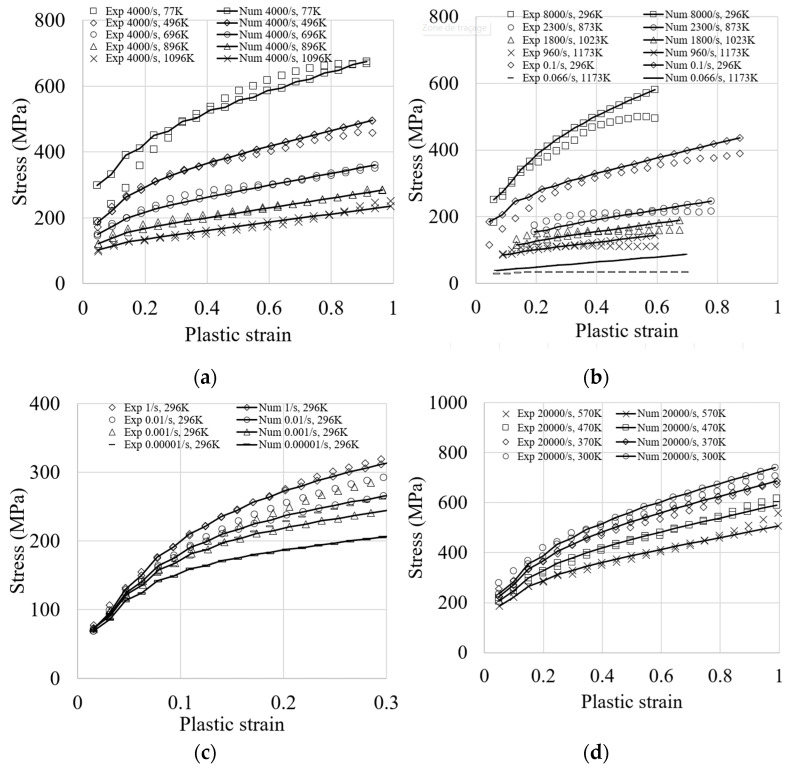

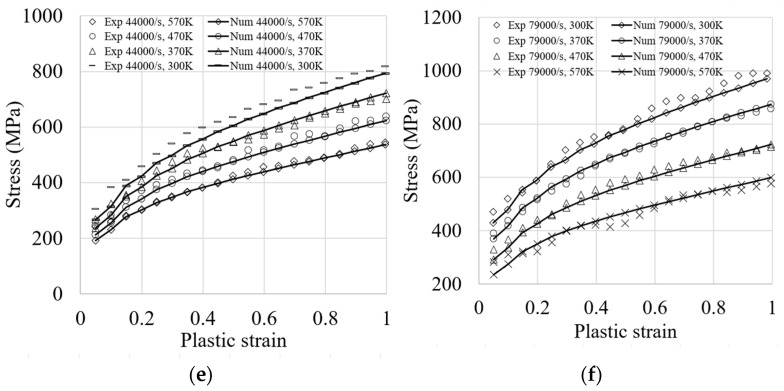
(**a**) Compression stress–strain curves at a strain rate of 4000/s and different temperatures. Experimental results are taken from [88]. (**b**) Compression stress strain curves at different temperatures and from very low to high strain rates up to 8000/s. Experimental results are taken from [85,86]. (**c**) Quasi-static compression tests at different strain rates and associated predicted responses. Experimental results are taken from [89]. (**d**) Compression stress–strain curves at a very high strain rate of 20,000/s and different temperatures. (**e**) Compression stress–strain curves at a very high strain rate of 44,000/s and different temperatures. (**f**) Compression stress strain curves at a very high strain rate of 79,000/s and different temperatures. Experimental results are taken from [90].

$N_{d}$ and $N_{p}$ are the number of simulated tests and the number of data points in each test, respectively. $\sigma_{exp}$ and $\sigma_{num}$ are the experimental and predicted flow stresses, respectively. The databases used in the automated minimization process are available in [86,88,89,90]. The calibrated set of material parameters is given in Table 2. The results of the calibration are illustrated in Figure 1. A good fit is obtained between the experimental database and the simulation results, which accurately describe the strain-rate sensitivity at different strain rates and temperatures. For instance, it can be observed from the fitted curves that the material becomes more sensitive to strain-rate variation where, at a given plastic strain of 1, the peak stress increases from about 700 MPa (4000/s) to 1000 MPa (79,000/s). Furthermore, the model has better capabilities in describing the material hardening compared to other existing models, which are assessed using the same experimental database, such as the Johnson–Cook, Steinberg–Cochran–Guinan–Lund, Zerilli–Armstrong, Mechanical Threshold Stress, and Preston–Tonks–Wallace plasticity models [85]. There are, however, some limits of the model in mimicking the hardening regime of copper at very high strain rates (8000/s) and low temperatures (296 K), which are due to the dependence of the yield stress to the initial dislocation density, and hence, to the initial and unknown microstructure configuration of the tested samples. In addition, softening due to the damage mechanism is not accounted for in the model. The strong coupling of the constitutive equations to a damage model would lead to better agreements.

**Figure 2:**
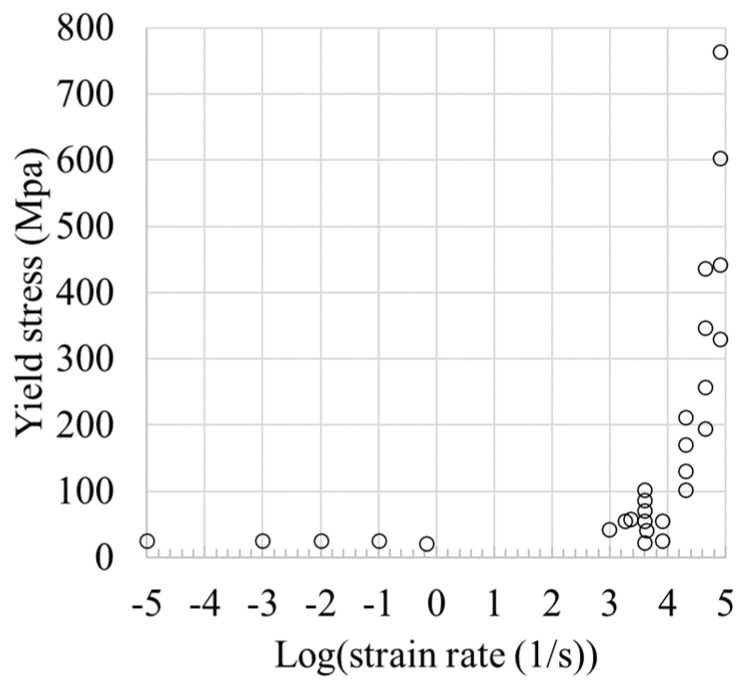
Yield stress at different strain rates. Stress strengthening was observed at a strain-rate transition of about 1000/s.

Another interesting aspect is the evolution of stress over an extended range of strain rates. Particular attention is dedicated to the ability of the model to describe the stress upturn in the viscous drag regime of dislocations. Hence, we assessed the stress strengthening at a varying strain rate from the stress–strain rate plot. In Figure 2, all the data points of the present study are inserted to observe the overall trend. As seen in the figure, the yield stress is almost independent of strain rate at strain rates below 1000/s. Above this value, the yield stress increases dramatically due to the transition of the deformation mechanisms from thermally activated to viscous drag-assisted deformation.

### 2.5. Finite Element Model

We conducted numerical simulations of single-particle impacts using the Abaqus2022/Explicit finite element code. The finite element model incorporated a spherical copper particle with a diameter (D) of 60 μm colliding with a semi-infinite copper substrate measuring 30D × 10D mm, at an incident angle of 90° and velocities ranging from 400 to 800 mm/s. For the sake of simplicity, we did not account for variations in impact angle in this study, although previous research has demonstrated its influence on bonding [18,38,91]. Both the particle and the substrate were treated as deformable bodies. The deviatoric behavior of the materials was modeled using the dislocation density-based approach, while the contribution of hydrostatic behavior was derived from the equation of state (EOS). However, a limited number of simulations were conducted with pure deviatoric stress (utilizing isotropic elasticity for the reversible deformation, denoted as ISO) to assess differences and observe its impact on overall behavior. We employed eight-noded, thermally coupled, linear brick elements (referred to as EC3D8RT) with reduced integration and hourglass control for the Eulerian mesh. In these simulations, the finite element model is constructed using a fine mesh with an element size of D/50. This choice was made based on various simulations of particle impact, as it minimizes mesh size effects and accurately captures deformation-related phenomena at the interface. The volume fraction of the elements was set to 1. To ensure periodic conditions on the substrate sides, non-reflecting Eulerian boundary conditions were applied to the impact-free surfaces of the substrate [38]. Furthermore, the substrate was made sufficiently large to eliminate any boundary effects arising from shock-wave propagation and reflection. The total simulation time was set at 30 ns, which was deemed adequate for stabilizing strain energy and achieving system equilibrium. The simulations were conducted under both coupled thermal mechanical (TM) and adiabatic (AD) conditions for this short impact duration, allowing us to investigate whether thermal energy dissipation significantly influenced deformation behavior. As a result, four main scenarios were examined, considering the influence of hydrostatic stress (EOS and isotropic elasticity) and the impact of thermal energy dissipation (adiabatic and coupled TM simulations). Subsequently, we illustrate the effects of shear viscosity and drag coefficient on deformation mechanisms, stress distribution, and dislocation density.

## 3. Results and Discussions

**Figure 3:**
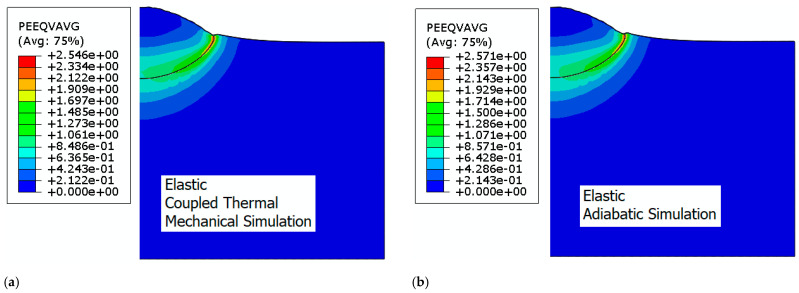

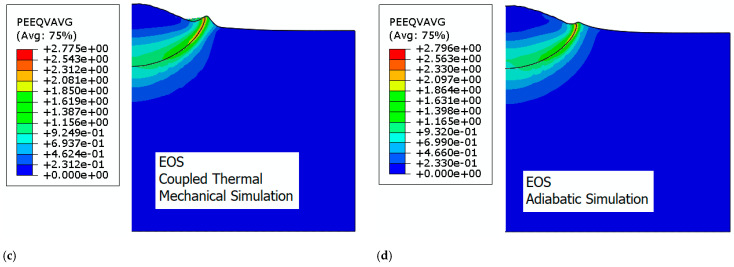
The deformation of the copper particle upon colliding with the substrate. (**a**) Simulations with thermal dissipation and isotropic elasticity. (**b**) Adiabatic simulations with isotropic elasticity. (**c**) Simulations with thermal dissipation and hydrostatic pressure consideration and (**d**) adiabatic simulations with hydrostatic pressure consideration.

In these finite element simulations, we initially examined the influence of hydrostatic stress through consideration of EOS and thermal energy dissipation on the deformation behavior of the particle–substrate assembly. The chosen initial velocity for the particle was 650 m/s, a value commonly associated with the critical velocity for copper bonding. The different deformed shapes of the particles are summarized in Figure 3. 

The simulations presented above illustrate the various shapes of the deformed particle. The simulations give a reasonable deformed shape of the particle in comparison with the experimentally observed deformed particle shapes [25]. Specifically, Figure 3a,b showcases the particle’s deformation when the elastic regime of the material is isotropic, and no EOS is accounted for. Figure 3c,d illustrate the results when EOS is considered. As demonstrated in Figure 3a,b, the particle remains confined within the substrate, and the inclination of the substrate material at the rim is minor. A negligible quantity of material arises from the interface of contact. The substrate deforms, creating a mechanical hooking along the rim. Conversely, in Figure 3c,d), the particle undergoes significant deformation at the contact surface with the substrate, causing material from both parts to displace towards the border. Notably, the substrate material at the border forms a circular bead with an inclination that can vary considerably. Specifically, for thermal mechanical simulation coupled with EOS (Figure 3c), which is the scenario that is most likely to occur, there is no peripheral hooking as observed for the other cases, which suggests the occurrence of another mechanism for adhesion. The particle cross-section presents a wavy shape which is not distinguishable when isotropic elasticity is considered to highlight the effect of the hydrostatic stress on deformation. This inclination serves as an indicator of the deformation intensity, and it is exacerbated by the elevated temperature experienced during impact.

**Figure 4:**
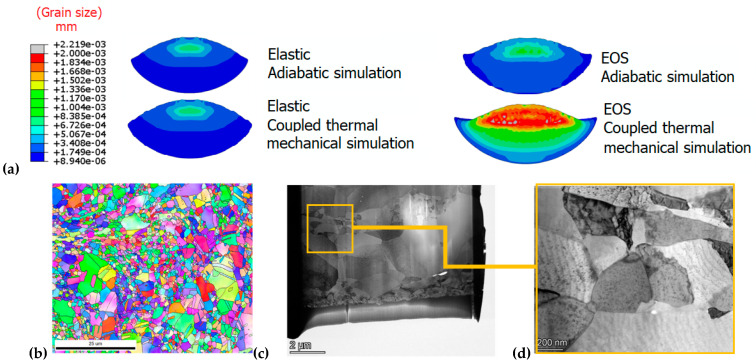
Grain size distribution in the deformed particle upon impact. (**a**) Different simulation cases were performed to depict the effect of hydrostatic pressure and temperature. (**b**) EBSD-IPF image of grain structure in CS copper coating; (**c**) TEM thin-foil showing the mixture of nano- and micro-scale grains; and (**d**) a TKD analysis of the IPF map. Reprinted/adapted with permission from Ref. [92], 2025, Elsevier.

**Figure 5:**
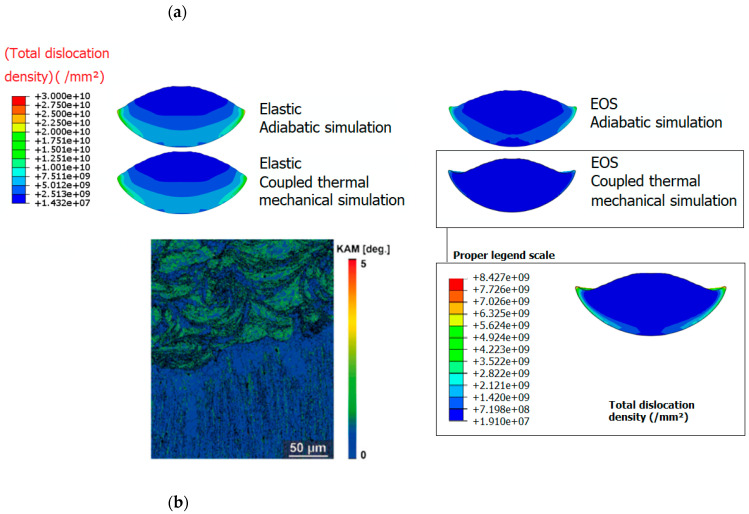
Total dislocation density distribution in the deformed particle upon impact. (**a**) Different simulation cases were performed to depict the effect of hydrostatic pressure and temperature. (**b**) KAM image indicating the dislocation density. Reprinted/adapted with permission from Ref. [94], 2025, Elsevier.

Figure 4a shows the grain size distribution in the deformed particle obtained by numerical simulations. In contrast to the other scenarios where the entire particle undergoes severe plastic deformation, grain refinement in the case of coupled thermal–mechanical simulation and EOS mostly occurs at the particle’s calotte, resulting in a surrounding area with a small grain size. Therefore, the role of hydrostatic stress seemingly promotes shearing and grain refinement in the periphery of the particle rather than achieving a diffuse refinement and an even distribution of plastic deformation throughout its entire body. The grain refinement at the particle substrate interface agrees well with the experimental observations [92,93]. Indeed, in a bigger scale, an overview observation in Figure 4b reveals grains with a random orientation, exhibiting a mix of ultra-fine and coarse sizes. This analysis shows that the CSAM Cu features a bimodal equiaxed grain structure, consisting of ultra-fine nano-grains ranging from 200 to 500 nm and micro-grains approximately 1 μm in size; the findings are available in [92]. This is in agreement with the grain sizes predicted numerically and ranging between 100 and 300 nm. These observations are strengthened by TEM observations and TKD analysis in Figure 4c,d, where micro-grains with no dislocation defects can be found surrounded by the typical equiaxial feature with grain sizes of 100–200 nm. Consequently, there is an impact on the dislocation density distribution. Numerical simulations shows that the total dislocation density is highest in the interparticle region, as shown Figure 5a, and only minimal microstructural changes occur at the particle core. The experimental results, such as the KAM map in [94], exhibit high KAM values in the particle interfaces in the copper coating, reflecting a higher dislocation density. As a result, adiabatic simulations provide higher temperatures, leading to a diffuse plasticity and an even distribution of the grain size over the particle, while the thermomechanical simulation caused a lower temperature due to heat transfer (EOS and thermomechanical simulation), thus promoting dislocation multiplication and strain hardening at the particle periphery.

**Figure 6:**
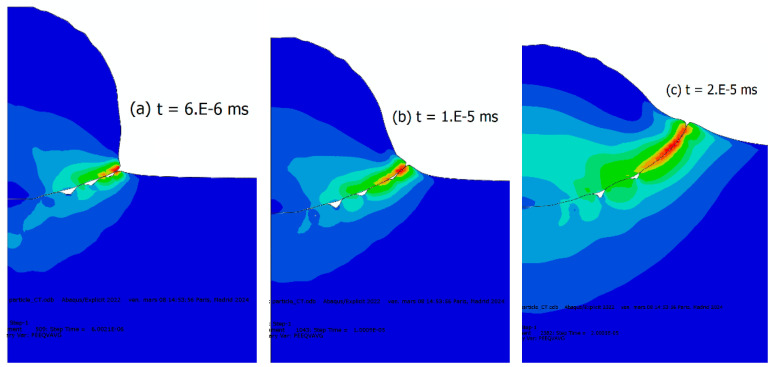
Plastic strain and waves observed during impact and their evolution with time for the coupled thermal mechanical case with EOS. (**a**) Early stages of the impact at t = 6 × 10^−6^ ms; (**b**) t = 1 × 10^−5^ ms; and (**c**) t = 2 × 10^−5^ ms.

**Figure 7:**
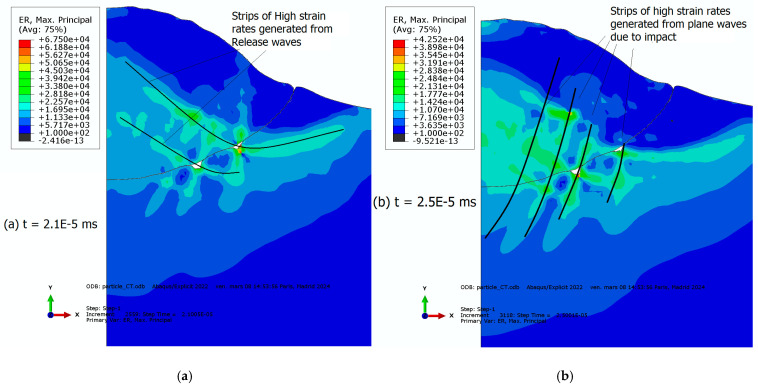

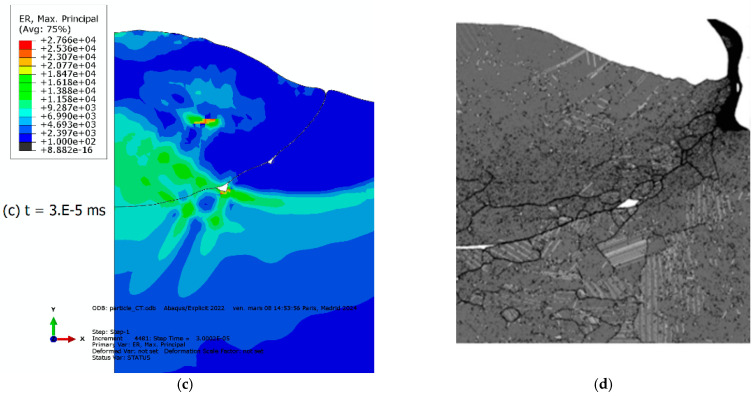
Strain-rate maps showing the propagation of the shock waves through the particle and substrate and the formation of material instabilities due to their interaction. (**a**) t = 2.1 × 10^−5^ ms; (**b**) t = 2.5 × 10^−5^ ms; and (**c**) t = 3.5 × 10^−5^ ms. (**d**) A real copper particle impacting a copper substrate. Reprinted/adapted with permission from Ref. [96], 2025, Elsevier.

To delve deeper into the bonding occurrence between the particle and substrate, we conducted additional simulations using a one-element-thickness model [95]. We assumed the same input parameters and boundary conditions as the 3D model described previously. Particular attention will be given to the deformation behavior at the particle–substrate interface for the coupled thermal–mechanical case and EOS consideration. Figure 6 shows the evolution of deformation over time at the particle–substrate interface. A wavy shape can be observed on the substrate side, continuously produced. This wavy shape is exclusively observed in the substrate, as the particle material is more confined in the crater, preventing excessive interfacial deformation from the particle side at the involved high-pressure levels. Therefore, the material deforms differently through the interface due to a disparity of strain-rate levels. These strain-rate levels are monitored by both the plane wave due to the impact and the release wave. Indeed, according to Figure 6, these material instabilities present as interface irregularities and form interfacial gaps (Figure 6) which are located at the intersection between the plane and release waves which can also be seen in Figure 7a–c at different time steps, progressing hand-in-hand during the earliest stage of the impact. It can also be seen that the material’s wavy shape does not initiate at the center of the impact area, suggesting that the oblique orientation of the plane waves, promoting shear behavior, is a precursor of the instabilities. This phenomenon was observed in a real-world cold-spray process and was demonstrated through molecular dynamics simulations where a gap is observed between the particle and the substrate [96], see Figure 7d for instance.

### 3.1. Shear Viscosity Effect

When the temperature approaches the melting point of the material, it exhibits fluid-like behavior, causing the plastic wave front to advance to the contact surface border and sporadically expel particle material to that region. Therefore, its effect is expected to be similar to the mechanical material softening due to excessive plastic deformation and damage development. In this section, we will focus on the effect of shear viscosity on the appearance of instabilities and material deformation. As mentioned earlier, the present study will use the simultaneous preheating of the both particle and substrate through different initial values of temperature, leading some of those cases to reach the viscosity threshold, above which the material behaves as a fluid-like Newtonian. In the present section, the temperatures chosen to exacerbate this effect are 500 K, 800 K, 1000 K, and 1200 K for the parts considered. The shear viscosity drastically but quasi-linearly decreases with increasing temperature. Consequently, the cohesion stress decreases, leading to a material more favorable to fluid flow. In all cases, even though shear viscosity is high at room temperature, the corresponding stress is still substantially lower than that generated by material hardening, unless the material experiences significant heating dissipation generated by plastic deformation.

**Figure 8:**
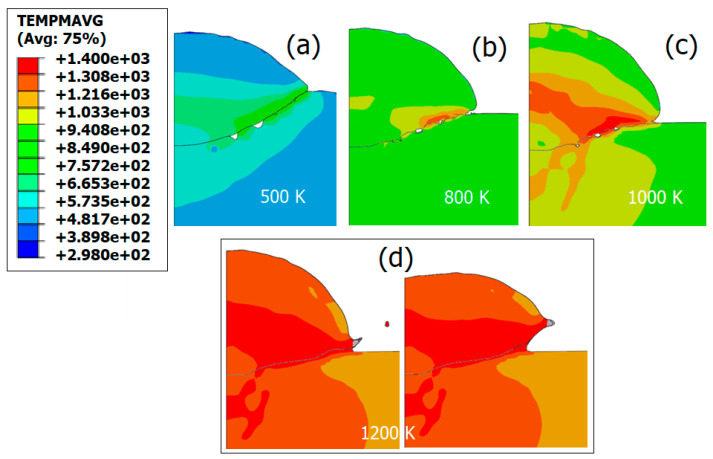
Shear viscosity effect on particle and substrate deformation (initially both at room temperature) for an adiabatic simulation with hydrostatic pressure consideration. Both substrate and particle are preheated at (**a**) T = 500 K; (**b**) T = 800 K; (**c**) T = 1000 K and (**d**) T = 1200 K (jetting produced and expelled far from the assembly).

**Figure 9:**
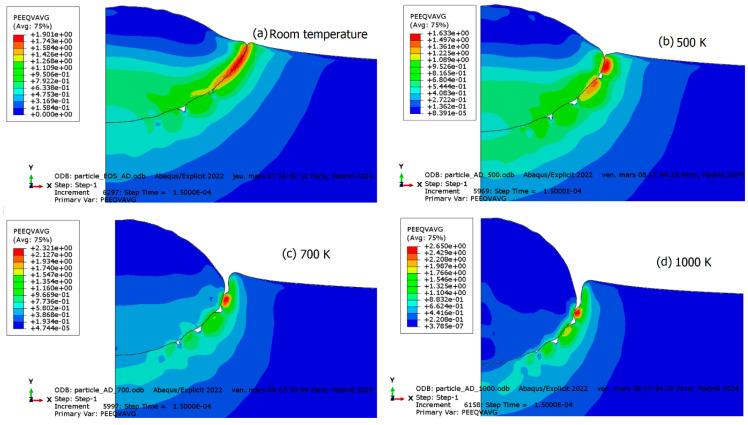
The deformation and morphology of the assembly when the substrate is preheated at different temperatures. (**a**) RT; (**b**) 500 K; (**c**) 700 K; and (**d**) 1000 K.

**Figure 10:**
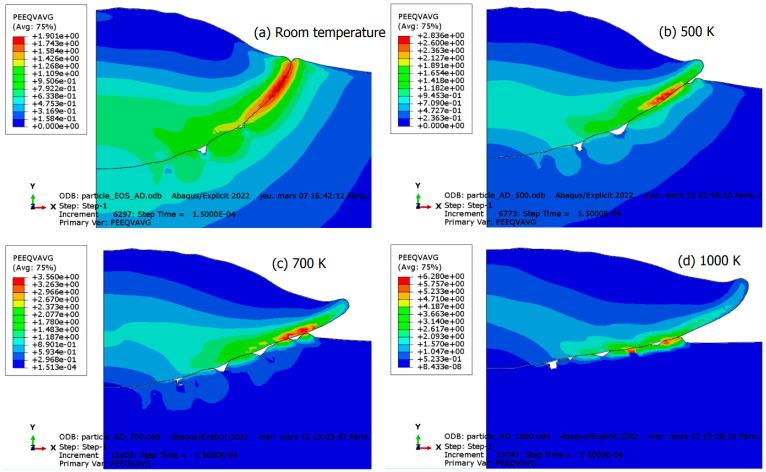
The deformation and morphology of the assembly when the particle is preheated at different temperatures. (**a**) RT; (**b**) 500 K; (**c**) 700 K; and (**d**) 1000 K.

The decrease in shear viscosity results in thermal material softening. Figure 8 illustrates the deformation of the particle and substrate assembly. It is evident that the deformation significantly increases when the viscosity threshold is reached earlier by imposing higher initial temperature to the parts considered. Material jetting occurs in the latter scenario at an early stage of impact due to the temperature rising beyond the viscosity threshold. Instabilities at the particle–substrate interface are apparent due to the difference in flow rates. The viscosity effect manifests as a drop in stress, thereby filling gaps in the particle–substrate interface with particle material, promoting intimate contact between the two, as depicted in Figure 8c. The latter scenario is likely to occur in case of extreme preheating of the particle and the substrate, as can be seen in some high temperature processes such thermal and plasma spraying. However, the scenario underscores the importance of viscosity in particle deformability, as it influences stress reduction and thermal softening. To further demonstrate the viscosity effect, additional simulations are conducted with preheated particle or substrate temperatures of 500 K, 700 K, and 1000 K (see Figure 9). In the case of a preheated particle, higher temperatures closer to the viscous threshold result in the severe flattening of the particle, as can be seen in Figure 10. This phenomenon is commonly observed in cold spray when soft particles deform on a hard substrate, either by using a different combination of materials [97] or softening one of the two materials by a preheating. Shear instabilities are also observed at the particle–substrate interface for all scenarios. However, an increase in particle temperature leads to a transition from uniform plastic deformation to localized regions with peaks of plastic deformation near the contact surface border. Furthermore, preheating the substrate leads to deeper particle penetration associated with an extended instability region at the interface as can be seen in Figure 9. Again, this is similar to a hard particle–soft substrate impact, such as in a copper–aluminum material system [98].

### 3.2. Drag Coefficient Effect

**Figure 11:**
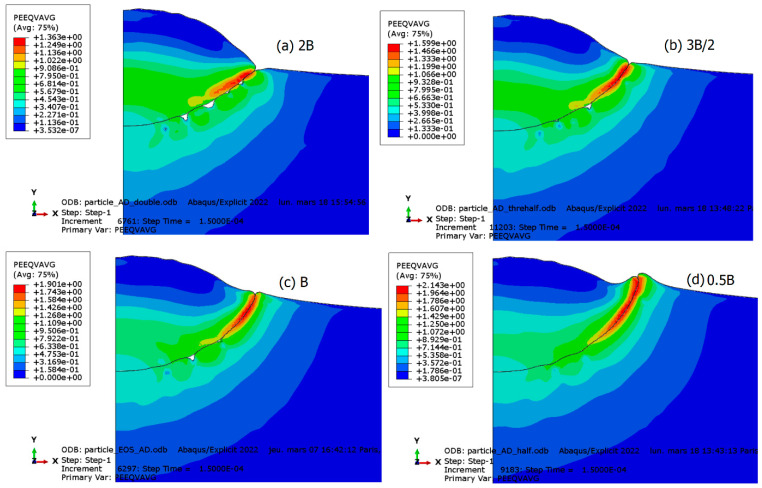
The influence of the drag coefficient B on the deformation and interfacial behavior of the particle and substrate. (**a**) 2B; (**b**) 3B/2; (**c**) B; and (**d**) 0.5B.

In the dislocation drag regime, which characterizes strain-rate hardening, viscous drag forces act on mobile dislocations. The drag coefficient, which relates stress to strain rate, plays a particularly significant role in this regime. Therefore, under conditions of very high strain rates, changes in this coefficient are expected to have a drastic impact on particle deformation and interfacial behavior. To assess its effect, several finite element simulations of particle impact have been conducted using different values of the drag coefficient, denoted as B. Specifically, values of 0.5B, B, 1.5B, and 2B were chosen. The results of plastic deformation are depicted in Figure 11. An analysis of the figure reveals that an increase in the drag coefficient leads to greater interfacial stability and reduced particle flattening. This observation can be attributed to the tight relationship between the drag coefficient and yield stress, which ultimately governs material hardness. Consequently, an increase in yield stress minimizes plastic deformation, particularly at the particle–substrate interface. This finding underscores the critical role of the drag coefficient in dictating the mechanical response of the system under high strain-rate conditions.

### 3.3. Adiabatic Shear Instability and Localization

In addition to the thermal softening associated with viscosity variation, a mechanism of shear instability may involve material damage following plastic deformation. The onset of damage results in material softening, which accelerates in the later stages of deformation, leading to strain localization. By considering damage evolution from a certain point, it is then possible to depict the occurrence of such a mechanism more clearly. A damage initiation and evolution law is considered hereafter [99,100]. The model considers fracture strain calculation for each deformation increment. The fracture strain depends on the stress triaxiality $\frac{\sigma_{m}}{\sigma_{mises}}$, the strain rate, and the temperature, and it is written as:

(31) $$\varepsilon_{f}=\left(D_{1}+D_{2}exp\left(D_{3}\frac{\sigma_{m}}{\sigma_{mises}}\right)\right)\left(1+D_{4}ln\left(\frac{\dot{\varepsilon }}{{\dot{\varepsilon }}_{0}}\right)\right)\left(1+D_{5}\frac{T-T_{ref}}{T_{m}-T_{ref}}\right)$$

$D_{1}$ to $D_{5}$ are all damage constants which are given for copper as 0.54, 0.28, 3.03, 0.014, and 1.12. Damage evolution is then deduced using:

(32) $$\;\;D=\sum \left(\frac{∆\varepsilon_{p}}{\varepsilon_{f}}\right)$$

**Figure 12:**
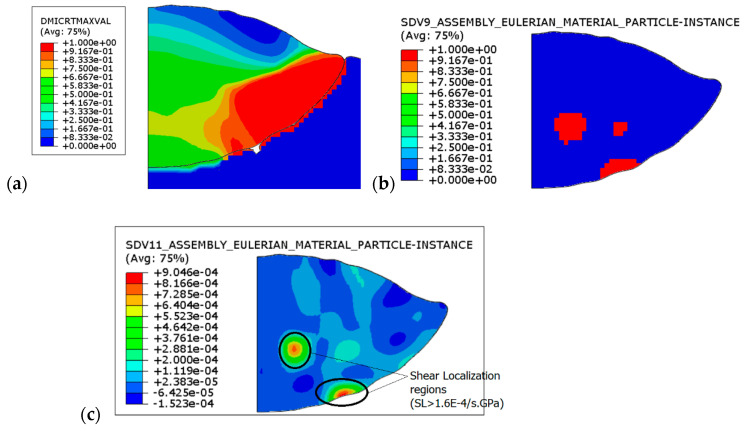
(**a**) The damage initiation criterion; (**b**) activated shear localization zones in the particle; and (**c**) the corresponding shear localization factor SL.

The shear localization criterion, represented by the shear localization factor SL, can then be monitored to depict the regions where shear localization occurs. By considering damage initiation and evolution in the new simulations, the areas of shear localization can be clearly identified. In Figure 12, we display three important quantities: the damage initiation criterion, shear localization areas, and shear localization factor SL. Firstly, the extent of shear instability has been previously described at the particle–substrate interface. Clearly, shear localization occurs at the shear instability region (interface), where the substrate displays severe surface discontinuity (Figure 12b). This occurrence of shear localization is crucial for particle–substrate bonding and necessary for mechanical interlocking between both surfaces. As mentioned earlier, the most common feature of shear localization is the formation of rollups in the shear instability region at the particle–substrate interface, especially for hard/soft material combinations. There is a second shear localization region inside the particle itself that does not affect the interface properties. Nevertheless, the SL in this region is caused by high stress levels and may constitute a source of shear band formation and crack initiation inside the particle, leading to substructure formation and particle fragmentation or splitting. Another research framework will be dedicated to the latter in a separate paper.

On the other hand, the damage initiation criterion shown in Figure 12a indicates material softening at the rim of the contact surface between the particle and the substrate. Again, this thermal material softening is favored by an increase in temperature, which, in turn, accelerates damage initiation and propagation. Additionally, thermal softening enhances metallurgical phenomena, leading to localized material intermixing. Associated with adiabatic shear instability, thermal material softening produces material jetting. As evidence of this feature, the observed material jetting is not continuous but sporadic. This observation could be clearer when shear viscosity is modified, for instance.

**Figure 13:**
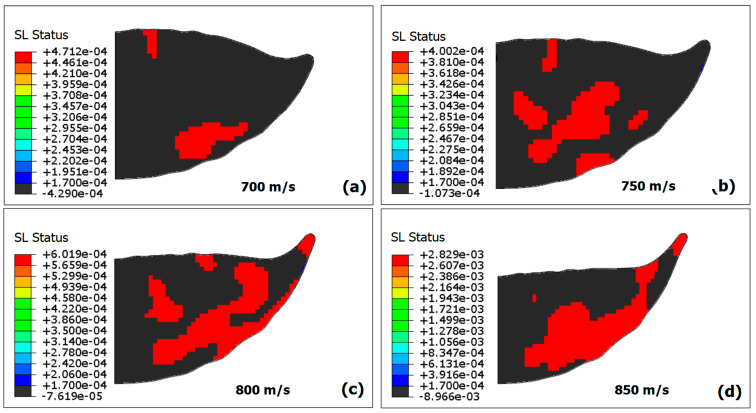
The effect of impact velocity on the SL region extent in the particle. (**a**) 700 m/s; (**b**) 750 m/s; (**c**) 800 m/s; and (**d**) 850 m/s.

Furthermore, the effect of impact velocity on shear localization is illustrated in Figure 13. With increasing particle velocity, more flattening and contour deformation occur. Shear localization extends to the particle interior with increasing velocity, forming intercrossing shear localization bands favorable to particle fragmentation. At the particle–substrate interface, the localization region tends to be larger with velocity and progressively deviates towards the particle rim.

## 4. Conclusions

To meet the growing demand for physically based models capable of (i) describing material behavior, (ii) capturing microstructural changes under extreme loading conditions of strain rate and temperature, and (iii) accurately predicting shear instability regions where bonding occurs at the impact interface between a particle and a substrate, we propose a plasticity model in this paper. This model is based on both comprehensive discrete dislocation dynamics (DDD) and a physical foundation of dislocation density evolution theory. The formulation of this model is detailed, and the significance of its various components is discussed. With material parameters calibrated from static and dynamic compression data, we demonstrate that the model can describe the stress–strain behavior of copper across a wide range of strain rates and temperatures. However, it should be noted that the model’s performance is influenced by the initial state, as justified by previous literature. Nevertheless, the model accurately reproduces features observed in experimental observations and successfully accounts for stress strengthening at the transition between thermally activated and viscous drag regimes. By employing this model to simulate the impact of copper particles onto a substrate, valuable insights into grain size and dislocation density in both the particle and substrate can be obtained. The model reveals that the initiation of the plastic shock wave is triggered by the hydrostatic stress that arises at the onset of particle impact. This plastic shock wave induces the formation of adiabatic shear instability regions at the particle–substrate interface, which are identified through finite element simulations. Furthermore, shear localization occurring at the particle–substrate interface is observed thanks to shear instability criteria and the shear localization factor coupled with the model. These shear localization regions, after shear instabilities, serve as foci for bonding through mechanical interlocking mechanisms. Therefore, elucidating bonding mechanisms at the particle–substrate interface using this model is feasible. Additionally, specific features such as material jetting and particle flattening are observed. For instance, shear viscosity plays a critical role in influencing material deformation, particularly at high temperatures nearing the material’s melting point. As the temperature increases and the material transitions toward fluid-like behavior, shear viscosity decreases, leading to thermal softening. This reduction in viscosity facilitates greater particle deformation and more pronounced material jetting at the particle–substrate interface. High shear viscosity at lower temperatures resists deformation, resulting in reduced material flow and a more constrained interface, which can limit bonding efficiency. The interplay between shear viscosity and temperature underscores its importance in controlling plastic deformation and optimizing particle–substrate contact during cold-spray processes. On the other hand, the drag coefficient is directly related to the resistance experienced by dislocations, significantly influencing strain-rate hardening and overall particle deformation during high-speed impact. An increase in the drag coefficient leads to higher yield stress, reducing plastic deformation and enhancing interfacial stability. This effect suppresses the excessive flattening of the particle and mitigates interface irregularities, potentially improving bonding uniformity. Conversely, a lower drag coefficient results in greater plastic deformation, which can lead to interfacial instability but may also enhance mechanical interlocking if controlled within optimal parameters. A more comprehensive exploration of the interactions between multiple parameters such as the shear viscosity, drag coefficient, and material temperature would provide deeper insights into the process and closer alignment with actual working conditions. The interplay between material softening due to thermal effects, damage generation, and adiabatic shear instability constitutes the origins of a distinct bonding mechanism, metallurgically based at the calotte border, which may lead to material intermixing and contribute to jetting and material expulsion from the interface.

## Figures and Tables

**Table 1:** Thermophysical properties of copper.

T (K)	ρ (kg.m^−3^)	Cp (J Kg^−1^ K^−1^)	λ (W m^−1^.K^−1^)	α	E (GPa)
300	8960	385	395	16 × 10^−6^	115
1300	8420	700	335	23 × 10^−6^	52

**Table 2:** Calibrated material parameters of copper using data from [85,89,90].

$\hat{\gamma }$	**0.01**	${\dot{\gamma }}_{0}$ **(s^−1^)**	**15**	***α***	**0.25**
$\alpha^{*}$	1.5 × 10^−5^	***Dp*** **(µm)**	20	β	0.225
$\beta_{i}$	0.00283	***K_inf_***	10	$k_{I1}$	−4.25 × 10^−5^
$f_{0}$	51.6	***K_0_***	10	$k_{w1}$	−4.2 × 10^−5^
$f_{\infty }$	0.0225	$\rho_{w0}$ **(mm^−2^)**	4622	$k_{I2}$	0.0081
***b*** **(nm)**	0.257	$\rho_{I0}$ **(mm^−2^)**	6320	$k_{w2}$	0.015
***ω_1_***	0.1	***A***	1.75 × 10^5^	$k_{i3}$	−0.2445
$\beta_{\infty }$	0.0086	k	1	$k_{w3}$	−5.38

## Data Availability

The original contributions presented in this study are included in the article. Further inquiries can be directed to the corresponding author.
